# Laparoscopic versus opengastric surgery for the treatment of pathological T_1_N_0_M_0_ gastric cancer in elderly patients: a matched study

**DOI:** 10.1038/s41598-017-02182-5

**Published:** 2017-05-15

**Authors:** Haiyan Pan, Tao Li, Zhigang Huang, Haibing Yu, Danli Kong, Yuanlin Ding, Congcong Pan, Yugang Jiang

**Affiliations:** 1 School of Public Health, Guangdong Medical University, Dongguan, 523808 Guangdong PR China; 2 Department of Chemotherapy, The People’s Hospital of Gaozhou, Gaozhou, 525200 Guangdong PR China; 3 Research Institute of The Aged Care Industry, Guangdong Medical University, Dongguan, 523808 Guangdong PR China; 4 Department of Gastrointestinal surgery, Shandong Provincial Hospital Affiliated to Shandong University, No. 324, Jing 5 Road, Jinan, 250021 Shandong PR China

## Abstract

The aim of this study was to compare the outcomes of laparoscopic surgery (LAP) and open gastric surgery (OP) in early gastric cancer patients aged ≥70 years.We conducted a retrospectively analysis among patientswith pathological T_1_N_0_M_0_ gastric cancer,who underwent LAP or OP between January 1, 2001 and December 31, 2008. We identified a well-balanced cohort of 2,360 patients (1180 patients in each group). LAP has been shown to offer a superior perioperative results to OP, including lower blood loss, shorter time to oral intake, walk and bowel function recovery, shorter time of hospital stay, and less blood transfusion required. However, the intraoperative and postoperative complications, local recurrence, and metastasis didn’t show statistically significant differences between groups. The 5-year overall survival (OS), disease-free survival (DFS), and cancer-specific survival (CSS) were 60.1% vs.63.2%, 80.8% vs. 83.3%, and 87.6% vs. 89.5% in the LAP group and OP group, respectively. The hazard ratios (HR) for OS, DFS, and CSS were 1.09(95% confidence interval [CI]: 0.95–1.25; P = 0.215), 1.03(95% CI: 0.91–1.18; P = 0.636), and 1.07 (95% CI: 0.88–1.30; P = 0.484), respectively, compared LAP group with OP group. In conclusion, LAP is an acceptable alternative to OP in elderly patients with early gastric cancer.

## Introduction

Gastric cancer is the third leading cause of cancer-related death worldwide, and it is the second most common type of cancer in China, accounting for nearly 42% of all new gastric cancer cases in the world^1,2^. So far, curative resection has been considered to be the most important indicator of long-term survival for patients with gastric cancer. Laparoscopic gastric surgery (LAP) for early gastric cancer has gained wide acceptance in Western countries after it was first introduced in 1991^3,4^. LAP was introduced into clinical practice in China in 2000, and was gradually implemented and is now commonplace in China^5^. Several studies have shown oncological outcomes after LAP for early gastric cancer to be comparable with those after open gastric surgery (OP)^6–8^. Long-term data comparing postoperative results between LAP and OP in patients with gastric cancer in China are important, since Chinese patients account for a large proportion; however, relevant studies are limited. In addition, postoperative morbidities and mortalities increase with age in elderly patients^9^, therefore, older patients are rarely included in the randomized studies because of this increasing risk. Thus far, the important issue of whether the LAP can serve as well as open approach in elderly patients with gastric cancer remains to be elucidated.

In this study, the long-term oncological outcomes of LAP and OP were evaluatedin a large cohort of elderly patients with pathologically confirmed stage T_1_N_0_M_0_ gastric cancer.

## Results

**Figure 1:**
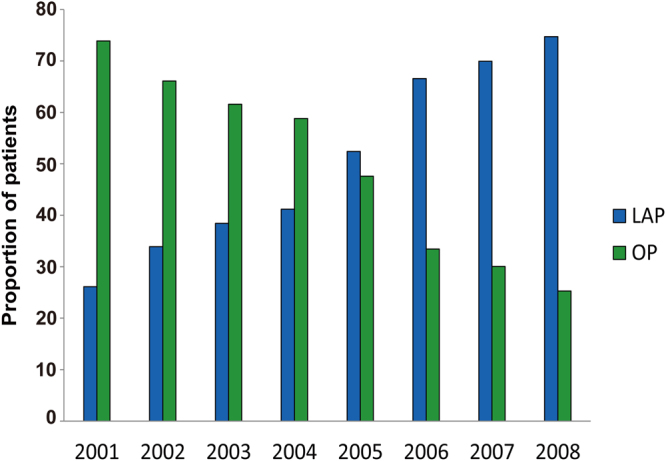
Rates of LAP and OP over time in patients with pathological stage T_1_N_0_M_0_ gastric cancer from the participating institutions.

Of the 4,786 patients with pathological stage T_1_N_0_M_0_ gastric cancer, 2,415 (50.5%) patients underwent LAP, and 2,371 (49.5%) underwent OP. Figure 1 shows that the proportions of LAP increased from 146/559 (26.1%) in 2001 to 473/633 (74.7%) in 2008. A total of 4,135 patients met the study inclusion criteria and were available for analyses in this study. Among them, 2,139 patients underwent LAP and 1,996 underwent OP. The characteristics of patients receiving LAP compared with those receiving OP are shown in Table 1. The LAP group was significantly older (P < 0.001), had a significantly higher BMI (P = 0.010), and had a significantly smaller tumor size (P = 0.033) compared to the OP group. In addition, significant differences were observed between the two treatment groups in terms of the distribution of sex (P < 0.001), ASA-PS class (P = 0.001), tumor site (P < 0.001), specific tumor stage (P = 0.032), histopathological type of tumor (P < 0.001), Lauren’s type of tumor (P < 0.001), surgical margins (P = 0.031), and postoperative chemotherapy (P = 0.019). After propensity score matching, all covariates were balanced and showed no statistically significant differences between the LAP group and OP group (Table 1). Consequently, we successfully matched 2,360 patients (57.1%) who received LAP (n = 1,180) or OP (n = 1,180) on the basis of the propensity score.

**Table 1:** Baseline demographics before and after propensity score matching.

Variable	Overall (n = 4135)			After propensity score matching (n = 2360)		
	Laparoscopic (n = 2139)	Open (n = 1996)	P value	Laparoscopic (n = 1180)	Open (n = 1180)	P value
Age (y, mean ± SD)	74.5 ± 7.2	73.2 ± 6.8	< 0.001	73.9 ± 7.0	73.6 ± 6.9	0.295
Sex (n, %)						
Male	1278 (59.7)	1078 (54.0)	<0.001	702 (59.5)	690 (58.5)	0.616
Female	861 (40.3)	918 (46.0)		478 (40.5)	490 (41.5)	
ASA-PS (n, %)						
1	768 (35.9)	621 (33.1)	0.001	385 (32.6)	362 (30.7)	0.347
2	912 (42.6)	868 (43.5)		521 (44.2)	556 (47.1)	
3	459 (21.5)	507 (25.4)		274 (23.2)	262 (22.2)	
BMI (kg/m^2^, mean ± SD)	23.9 ± 10.3	23.1 ± 9.7	0.010	23.5 ± 7.4	23.4 ± 7.1	0.738
Tumor size (mm, mean ± SD)	37.6 ± 12.4	38.4 ± 11.5	0.033	37.9 ± 9.1	38.2 ± 9.3	0.428
Tumor site (n, %)						
Lower segment	367 (17.2)	274 (13.7)	<0.001	202 (17.1)	195 (16.5)	0.723
Lower to middle segment	149 (6.9)	101 (5.1)		89 (7.5)	73 (6.2)	
Middle segment	806 (37.7)	735 (36.8)		427 (36.2)	438 (37.1)	
Middle to upper segment	235 (11.0)	241 (12.1)		156 (13.2)	164 (13.9)	
Upper segment	582 (27.2)	645 (32.3)		306 (26.0)	310 (26.3)	
Previous abdominal surgery (n, %)	234 (10.9)	193 (9.7)	0.180	119 (10.1)	113 (9.6)	0.678
Tumor stage (n, %)						
T_1a_N_0_M_0_	1455 (68.0)	1419 (71.1)	0.032	824 (69.8)	833 (70.6)	0.685
T_1b_N_0_M_0_	684 (32.0)	577 (28.9)		356 (30.2)	347 (29.4)	
Tumor grade (n, %)						
1	599 (28.0)	486 (24.3)	0.056	312 (26.5)	298 (25.3)	0.788
2	933 (43.6)	928 (46.5)		535 (45.3)	554 (46.9)	
3	545 (25.5)	527 (26.4)		305 (25.8)	296 (25.1)	
Unclassified	62 (2.9)	55 (2.8)		28 (2.4)	32 (2.7)	
Histopathological type (n, %)						
Papillary adenocarcinoma	651 (30.4)	496 (24.8)	<0.001	349 (29.6)	317 (26.9)	0.219
Tubular adenocarcinoma	507 (23.7)	568 (28.5)		286 (24.2)	326 (27.6)	
Poorly differentiated adenocarcinoma	296 (13.8)	281 (14.1)		166 (14.1)	174 (14.7)	
Mucinous adenocarcinoma	359 (16.8)	392 (19.6)		212 (18.0)	223 (18.9)	
Signet-ring cell carcinoma	215 (10.1)	163 (8.2)		115 (9.7)	95 (8.1)	
Others	111 (5.2)	96 (4.8)		52 (4.4)	45 (3.8)	
Lauren’s type (n, %)						
Intestinal	1222 (57.1)	1097 (55.0)	<0.001	670 (56.8)	647 (54.8)	0.149
Diffuse	813 (38.0)	734 (36.8)		450 (38.1)	451 (38.2)	
Mixed	104 (4.9)	165 (8.2)		60 (5.1)	82 (7.0)	
Local extent of gastric resection (n, %)						
Subtotal gastrectomy	1960 (91.6)	1800 (90.2)	0.104	1072 (90.8)	1063 (90.1)	0.528
Total gastrectomy	179 (8.4)	196 (9.8)		108 (9.2)	117 (9.9)	
Surgical margins (n, %)						
Negative	1887 (88.2)	1716 (86.0)	0.031	1027 (87.0)	1016 (86.1)	0.507
Positive	252 (11.8)	280 (14.0)		153 (13.0)	164 (13.9)	
Postoperative chemotherapy(n, %)						
Yes	93 (4.3)	96 (4.8)	0.019	53 (4.5)	56 (4.7)	0.714
No	1905 (89.1)	1808 (90.6)		1052 (89.2)	1058 (89.7)	
Unknown	141 (6.6)	92 (4.6)		75 (6.3)	66 (5.6)	
Postoperative radiotherapy(n, %)						
Yes	142 (6.6)	157 (7.9)	0.099	81 (6.9)	86 (7.3)	0.514
No	1928 (90.2)	1791 (89.7)		1060 (89.8)	1064 (90.2)	
Unknown	69 (3.2)	48 (2.4)		39 (3.3)	30 (2.5)	

ASA-PS, American Society of Anesthesiologists physical status; BMI, body mass index; SD, standard deviation.

The mean follow-up duration was 59.6 months for LAP group and 60.4 months for OP group. The intraoperative and postoperative results of the propensity score matched cohort are shown in Table 2. The operative time was significantly longer LAP group than in the OP group (P < 0.001). However, the estimated blood loss was significantly lower in the LAP group than in the OP group (P < 0.001). There were no significant differences in the number of lymph node dissection (P = 0.142) and the number of positive lymph nodes (P = 0.332). The mean times to oral intake after surgery were 2.3 days and 3.3 days (P < 0.001) for LAP group and OP group, respectively. It took a longer time for patients to walk (2.7 days vs. 1.4 days, P < 0.001) and bowel function recovery (2.8 days vs. 2.1 days, P < 0.001) after surgery in the OP group, compared with LAP group. The postoperative hospital stay was longer in the OP group (13.6 days vs. 11.2 days, P < 0.001).

**Table 2:** Intraoperative and postoperative results.

Variable	Laparoscopic (n = 1180)	Open (n = 1180)	P value
Follow-up duration (mo, mean ± SD)	59.6 ± 11.4	60.4 ± 12.6	0.106
Operative time (min, mean ± SD)	263.2 ± 58.7	184.5 ± 49.1	<0.001
Estimated blood loss (ml, mean ± SD)	149 ± 155	263 ± 232	<0.001
Number of lymph node dissection (mean ± SD)	27.3 ± 3.5	27.1 ± 3.1	0.142
Number of positive lymph nodes (mean ± SD)	8.4 ± 2.6	8.3 ± 2.4	0.332
Time to oral intake after surgery (d, mean ± SD)	2.3 ± 0.9	3.3 ± 1.2	<0.001
Time to walk after surgery (d, mean ± SD)	1.4 ± 0.5	2.7 ± 1.0	<0.001
Time to bowel function recovery (d, mean ± SD)*	2.1 ± 0.4	2.8 ± 0.7	<0.001
Postoperative hospital stay (d, mean ± SD)	11.2 ± 2.3	13.6 ± 2.9	<0.001
Blood transfusion required (n, %)	213 (18.1)	347 (29.4)	<0.001
Intraoperative complications (n, %)	102 (8.6)	113 (9.6)	0.431
Postoperative complications, ≥grade 3 (n, %)**	149 (12.6)	167 (14.2)	0.277
Local recurrence (n, %)	153 (13.0)	146 (12.4)	0.665
Metastasis (n, %)	102 (8.6)	97 (8.2)	0.711
Number of patients undergoing a curative second resection (n, %)	104 (8.8)	93 (7.9)	0.413

SD, standard deviation.

^*^Defined as time of anal exhaust or defecation.

^**^According to the Clavien-Dindo classification.

Intraoperative complications occurred in 102 (8.6%) patients in the LAP group and 113 (9.6%) patients in the OP group (P = 0.431) (Table 2). The most frequently occurred intraoperative complications were vascular hemorrhage and surgical injuries. The incidence of vascular hemorrhage and surgical injuries didn’t show significantly differences between two treatment groups (P = 0.249, and 0.113, respectively) (Table 3). The incidence of postoperative complications that were grade 3 or greater according to the Clavien-Dindo classification was 12.6% in the LAP group and 14.2% in the OP group, which didn’t show significant differences (P = 0.277) (Table 2). With regard to the specific postoperative complication, there were no significantly difference in intra-abdominal bleeding (P = 0.391), intraluminal bleeding (P = 0.519), anastomotic leakage (P = 0.652), pancreatic fistula (P = 0.281), abdominal abscess or fluid collection (P = 0.592), wound infection (P = 0.288), stenosis (P = 0.465), enteroparalysis (P = 0.0.511), ascites (P = 0.371), pneumonia (P = 0.365), cardiac problems (P = 1.000), and internal hernia (P = 0.617) between two groups; however, the LAP group had significantly lower risk of small bowel obstruction (P = 0.003), compared with the OP group (Table 3). Local recurrence occurred in 153 (13.0%) patients in the LAP group and 146 (12.4%) patients in the OP group, which didn’t reach statistical significance (P = 0.665) (Table 2). There were no significant differences in metastasis between two treatment groups (8.6% vs. 8.2%, P = 0.711) (Table 2). The most frequent metastasis sites were lymph node, ovary (for female patients), liver, lung, brain, and bone, and there were no significant differences on these sites between two groups (all P values > 0.05); however, the risk of peritoneal metastasis was significantly lower in the LAP group than that in the OP group (1.0% vs. 2.1%, P = 0.031) (Table 3). Moreover, there were no significant differences in number of patients undergoing a curative second resection between two groups (8.8% vs. 7.9%, P = 0.413) (Table 2).

**Table 3:** Results of complications and metastasis.

Variable (n, %)	Laparoscopic (n = 1180)	Open (n = 1180)	P value
Intraoperative complications			
Vascularhemorrhage	53 (4.5)	42 (3.6)	0.249
Surgical injuries	39 (3.3)	54 (4.6)	0.113
Others	18 (1.5)	27 (2.3)	0.176
Postoperative complications (≥grade 3)*			
Short-term problems			
Intra-abdominal bleeding	22 (1.9)	28 (2.4)	0.391
Intraluminal bleeding	42 (3.6)	48 (4.1)	0.519
Anastomotic leakage	24 (2.0)	21 (1.8)	0.652
Pancreatic fistula	18 (1.5)	25 (2.1)	0.281
Abdominal abscess or fluid collection	6 (0.5)	8 (0.7)	0.592
Wound infection	2 (0.2)	6 (0.5)	0.288
Stenosis	7 (0.6)	10 (0.8)	0.465
Enteroparalysis	9 (0.8)	12 (1.0)	0.511
Ascites	1 (0.1)	4 (0.3)	0.371
Pneumonia	4 (0.3)	7 (0.6)	0.365
Cardiac problem	2 (0.2)	1 (0.1)	1.000
Neurological problem	0 (0.0)	0 (0.0)	—
Others	7 (0.6)	12 (1.0)	0.249
Long-term problems			
Small bowel obstruction	12 (1.0)	31 (2.6)	0.003
Internal hernia	1 (0.1)	3 (0.3)	0.617
Postoperative metastasis site			
Lymph node	41 (3.4)	32 (2.7)	0.285
Ovary**	27 (5.5)	18 (3.8)	0.197
Liver	29 (2.5)	26 (2.2)	0.682
Lung	23 (1.9)	19 (1.6)	0.533
Peritoneum	12 (1.0)	25 (2.1)	0.031
Brain	15 (1.3)	11 (0.9)	0.430
Bone	8 (0.7)	11 (0.9)	0.490
Others	13 (1.1)	9 (0.8)	0.392

^*^According to the Clavien-Dindo classification.

^**^Calculated and compared only in female patients.

**Figure 2:**
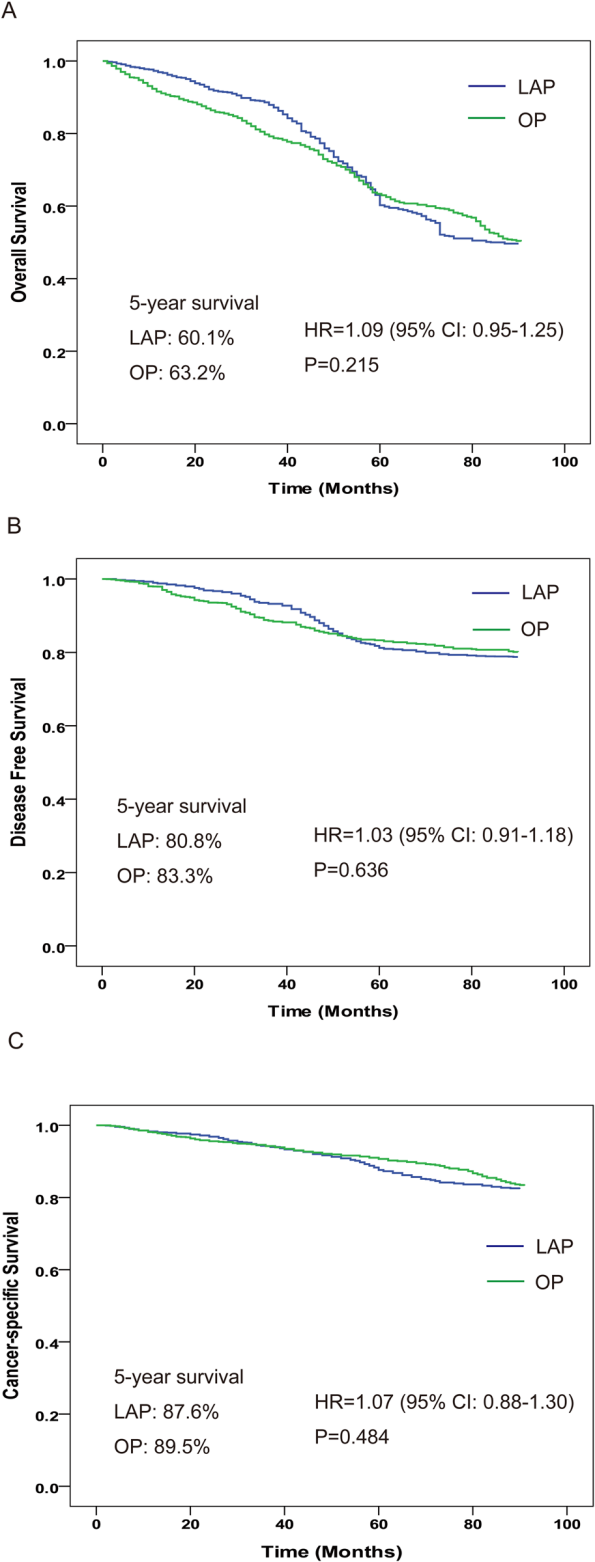
Overall survival rate (**A**), disease-free survival rate (**B**), and cancer-specific survival rate (**C**) in LAP and OP.

Figure 2 shows the survival curves obtained using the Kaplan-Meier method. The 5-year OS was 60.1% (95% CI: 58.2–62.0%) in the LAP group as compared with 63.2% (95% CI: 61.5–64.9%) in the OP group. In addition, the 5-year DFS was 80.8% (95% CI: 79.0–82.6%) in the LAP group and 83.3% (95% CI: 81.5–85.1%) in the OP group, and the 5-year CSS was 87.6% (95% CI: 86.1–89.1%) in the LAP group and 89.5% (95% CI: 87.9–91.1%) in the OP group. There were no significant differences between patients who underwent LAP and OP in terms of OS (HR = 1.09, 95% CI: 0.95–1.25; P = 0.215), DFS (HR = 1.03, 95% CI: 0.91–1.18; P = 0.636), and CSS (HR = 1.07, 95% CI: 0.88–1.30; P = 0.484).

## Discussion

Our study suggests that LAP offers a superior perioperative results to OP in elderly patients with pathological stage T_1_N_0_M_0_ gastric cancer, including lower blood loss, shorter time to oral intake, walk andbowel function recovery, and shorter time of hospital stay after surgery, and less blood transfusion required. However, there are no significant differences in intraoperative and postoperative complications, local recurrence, and metastasis between LAP and OP, and that there are no differences in long-term oncological outcomes as assessed by OS, DFS, and CSS. These results were mostly consistent with those obtained from the general population^10–12^ and those obtained from patients with advanced gastric cancer^13–15^. However, Lee and colleagues^10^ found that the postoperative complications of patientswith early gastric cancer in the LAP group occurred less frequently compared with in the OP group (4.7% vs. 13.3%, P = 0.046), which is different from our study (16.3% vs. 14.7%, P = 0.279). A meta-analysis^16^ conducted among the general population also indicated that laparoscopic technique was associated with lower surgical complications. Therefore, elderly patients who underwent LAP may have a higher risk of postoperative complications compared with the general population; however, more studies are needed to further confirm this inference since other baseline characteristics may be different between different studies.

To our best knowledge, this is the first large-scale cohort study to compare the therapeutic efficacy between LAP and OP, involving elderly patients (mean age of 73.9 in the matched LAP group and 73.6 in the matched OP group) with pathological stage T_1_N_0_M_0_ gastric cancer. Although a randomized controlled trial (RCT) would be a better choice to compare LAP and OP, the increasing risk of postoperative morbidities and mortalities with advancing age may limit the inclusion of elderly patients in the RCTs^17–19^. We used a propensity score matching method in our observational study to balance the covariates and control for the selection bias, and this method could simulate the conditions of RTCs^20–22^. As we had a large sample size and we performed strict propensity score matching between the two groups, the results of this study seem to establish that LAP is surgically favorable and oncologically comparable to OP for T_1_N_0_M_0_ gastric cancer in elderly patients.

From this study we can see that the proportion of patients who were treated with LAP for early gastric cancer had a consistent increase during the period of 2001–2008. This may be due to the fact that more and more patients and physicians realize that using the laparoscopic technique is less invasive and easier to recovery, and this is especially important for elderly patients. Our study shows the mean estimated blood loss was 149 ml in the LAP group and 263 ml in the OP group, and the rate of blood transfusion was 18.1% compared with 29.4%. A previous study^23^ compared the short-term outcomes of laparoscopic and open gastrectomy for gastric cancer and found the postoperative hospital stay was 9.5 days and 10.9 days for laparoscopic and open technique, respectively. The hospital stay after surgery was a little longer in both groups in our study (11.2 days vs.13.6 days) compared with theirs. However, the mean age of patients was much younger in their study (52.3 years vs. 54.3 years for laparoscopic and open technique, respectively). Thus, advanced age may delay the recovery and prolong the hospital stay.

Li and colleagues^24^ also compared the therapeutic effect of LAP and OP in elderly patients with gastric cancer, however, the sample size was quite small in their study (54 patients for each group). In addition, they only compared OS between the two groups and found that the 3-year survival rates were 55.6% and 57.4% for LAP and OP, respectively. As elderly patients are more likely to die from other causes (nearly half of the deaths were caused by cardiovascular accidents in their study), thus there would be an obvious decrease in OS during the follow up. Therefore, we also assessed the CSS in our study. We found that the 5-year CSS were 87.6% and 89.5% for LAP and OP, respectively, and there were no statistical differences in CSS between two groups. Although our study was an analysis among patients with pathological T_1_N_0_M_0_ early gastric cancer, the 5-year DFS and 5-year CSS were relatively low. This may be due to the following reasons: (1) the patients were relatively old (mean age of nearly 75 years old) in both groups at baseline, with poor physical fitness and immunity, which led to lower gastric cancer survival; and (2) China is a developing country, and the patients were mostly from the countryside, thus, the socioeconomic level of the patients was relatively low. Many studies^25,26^ have demonstrated that lower socioeconomic level was associated with poorer prognosis of gastric cancer.

The advantages of our study are that we had a much larger sample size compared with previous studies, we have a long-term follow-up duration, and we compared the results specifically in the elderly patients. However, there are several limitations in this study. Firstly, we used observational data, and although we performed rigorous propensity score matching between groups, unmeasurable orunknownconfounding factors are possible. A RCT would control for all possible confounders. However, conducting a RCT is impractical among elderly patients. Secondly, the degree of operation proficiency could affect the outcome of any comparison between LAP and OP. However, we were unable to adjust the operation proficiency in current study. It is impossible to assign surgeons with exactly the same level of skill to LAP group and OP group. Even in a RCT, it is also difficult to control for this factor. Usually, the same surgeon is either proficient in laparoscopic or open technique. Surgeons who perform both surgery types may be better in one or the other. Therefore, our findings should be interpreted with the consideration of operation proficiency, which may be an important source of bias. Thirdly, our study was conducted among patients with early gastric cancer, with mean tumor size smaller than 4 cm. LAP has been proved possible for gastric tumors lager than 5 cm^27^. However, patients with large tumors usually underwent OP or conservative treatment in the participating institutions, thus, we are unable to make a comprehensive comparison of LAP and OP in elderly patients with large gastric tumors. Fourthly, our study is limited by the amount of LAP and OP cases over time, since LAP is a newer technique and the amount has increased over time.

In conclusion, we found that in matched cohort of elderly patients with pathological stage T_1_N_0_M_0_ gastric cancer, LAP offers a superior perioperative results to OP, and LAP and OP were associated with similar long-term oncological outcomes. LAP is an acceptable alternative to OP in elderly patients with early gastric cancer.

## Methods

The study was supported by the Shandong Provincial Hospital institutional review board, with approval number as 2013–0021B. All methods were performed in accordance with the relevant guidelines and regulations.Informed consent was obtained from each patient.

### Patients

We identified all patients from six Chinese cancer-specialized institutions. To be included in the study, patients were required to have a clinical diagnosis of pathological stage T_1_N_0_M_0_ gastric cancer, age of at least 70 years, being able to offer either laparoscopic or open surgery data, and at least 36 months follow up. Exclusion criteria included the presence of other primary malignancies, a history of chemotherapy or radiotherapy before surgery, and American Society of Anesthesiologists physical status (ASA-PS) score >3. The presence or absence of coexisting conditions was assessed with the International Classification of Diseases, Ninth Revision, Clinical Modification (ICD-9-CM). Between January 1, 2001 and December 31, 2008, a total of 4,786 patients aged ≥70 years underwent surgery for pathological stage T_1_N_0_M_0_ gastric cancer at the participating institutions. However, 651 patients failed to meet the inclusion criteria and were ineligible for the study. The following baseline data were obtained for each patient: age, sex, ASA-PS score, body mass index (BMI), tumor size, tumor site, previous abdominal surgery history, specific tumor stage, tumor grade, histopathological type of tumor, Lauren’s type of tumor, surgical margins, and postoperative chemotherapy. The choice of the surgery approach was based on patient and physician preference. The performances of the laparoscopic and open techniques were strictly adhered to surgical oncological principles^28^. Considering the average number of gastric cancer patients undergoing gastric surgery was more than 400 cases per year during this period, all surgeons were considered to have enough experiences to perform both laparoscopic and open gastric surgery, although there were no surgeon-specific criteria in this study. The primary end point of our study was overall survival (OS). Secondary endpoints included disease-free survival (DFS), cancer-specific survival (CSS), postoperative recovery, and the incidence of postoperative complications that were grade 3 or greater according to the Clavien-Dindoclassification^29^.

### Statistical analysis

Summary statistics were constructed with the use of means and standard deviation (SD) for continuous variables and frequencies and proportions for categorical data. We retrospectively compared the demographic, intraoperative, postoperative and oncological follow up data between the two surgery groups. Student’s t test was used for continuous data and Chi-square and z tests were used for categorical data. The OS, DFS, and CSS were assessed using the Kaplan-Meier method and compared between the LAP and OP groups. Cox proportion hazards model was used to estimate the hazard ratios (HR) and 95% confidence intervals (CI).

To reduce the effect of treatment-selection bias and potential confounding in this observational study, we used the propensity score matching method to balance the observed covariates between the treatment groups. Each patient was assigned a propensity score by using a nonparsimonious logistic regression model that included all patient and hospital characteristics. Two patients with identical propensity scores included in the LAP group and in the OP group could be considered randomly assigned to each group, and balanced propensity score could theoretically lead to unbiased estimates of between-group differences^21^. A one-to-one matched analysis without replacement on the basis of the estimated propensity score of each patient was performed. Patients in the OP group who had an estimated logit within 0.6SD of the selected patients in the LAP group were eligible for matching, since 0.6SD has been shown to eliminate approximately 90% of the bias in observed confounders^30^.

All reported P values are two-sided. A P value of less than 0.05 was considered to indicate statistical significance.
