# Erratum: Laparoscopic versus opengastric surgery for the treatment of pathological T_1_N_0_M_0_ gastric cancer in elderly patients: a matched study

**DOI:** 10.1038/s41598-017-09607-1

**Published:** 2017-10-09

**Authors:** Haiyan Pan, Tao Li, Zhigang Huang, Haibing Yu, Danli Kong, Yuanlin Ding, Congcong Pan, Yugang Jiang

**Affiliations:** 1 School of Public Health, Guangdong Medical University, Dongguan 523808 Guangdong, PR China; 2 Department of Chemotherapy, The People’s Hospital of Gaozhou, Gaozhou 525200 Guangdong, PR China; 3 Research Institute of The Aged Care Industry, Guangdong Medical University, Dongguan 523808 Guangdong, PR China; 4 Department of Gastrointestinal surgery, Shandong Provincial Hospital Affiliated to Shandong University, No. 324, Jing 5 Road, Jinan, 250021 Shandong PR China

*Scientific Reports* **7**:1919; doi:10.1038/s41598-017-02182-5; Article published online 15 May 2017

In the original version of this Article, Affiliation 4 was incorrectly listed as ‘Department of Gastrointestinal Surgery, Shandong Provincial Hospital, Jinan, 250021, Shandong, PR China’. The correct affiliation is listed below.

Department of Gastrointestinal surgery, Shandong Provincial Hospital Affiliated to Shandong University, No. 324, Jing 5 Road, Jinan 250021, Shandong, PR China.

This error has now been corrected in the PDF and HTML versions of the Article.

